# Novel Reactive Polyhedral Oligomeric Silsesquioxane-Reinforced and Toughened Epoxy Resins for Advanced Composites

**DOI:** 10.3390/polym16131877

**Published:** 2024-07-01

**Authors:** Weibo Liu, Caiyun Wang, Yu Feng, Yongfeng Chen, Liqiang Wan, Farong Huang, Zuozhen Liu, Jianhua Qian, Weiping Liu

**Affiliations:** 1 Key Laboratory for Specially Functional Polymeric Materials and Related Technology of (Ministry of Education), School of Materials Science and Engineering, East China University of Science and Technology, Shanghai 200237, China; y82210301@mail.ecust.edu.cn (W.L.); y10210074@mail.ecust.edu.cn (C.W.); fengyu10161366@163.com (Y.F.); 13918350379@163.com (Y.C.); wanliqiang@163.com (L.W.); 2 HuaChang Polymers Co., Ltd., East China University of Science and Technology, Shanghai 200241, China; lzz@ecust.edu.cn (Z.L.); qjh@hchp.com.cn (J.Q.); 3 Manufacturing Center of Composite Materials for Commercial Aircraft, Shanghai Aircraft Manufacturing Co., Ltd., COMAC, Shanghai 201324, China; liuweiping@comac.cc

**Keywords:** high-performance epoxy resin, polyhedral oligomeric silsesquioxane, toughness, liquid molding resin for advanced composites

## Abstract

Most toughening methods for epoxy resins are usually used at the expense of other properties. Some polyhedral oligomeric silsesquioxanes (POSSs) with both a rigid Si-O-Si structure and flexible organic chain segments could be expected to be effective toughening agents. In this study, three reactive polyhedral oligomeric silsesquioxanes with a thiol group (OMPPS), a carboxyl group (OCOPS), and an epoxy group (OGCPS) were synthesized and characterized. They were utilized as modifiers to toughen 3-(oxiran-2-ylmethoxy)-*N*,*N*-bis(oxiran-2-ylmethyl)aniline (AFG-90MH)/4,4′-methylenebis(2-ethylaniline) (MOEA) (epoxy resin) with different molar ratios to obtain hybrid resins named OMPPS-EP-i, OCOPS-EP-j, and OGCPS-EP-k. The effects of the amount of modifier added and the length of the organic chain on the cage structure on various properties of the hybrid resins were investigated. The results show that all three modifiers show good compatibility with the epoxy resin. The hybrid resins have a low viscosity at 45~85 °C and can be cured at a low temperature (110 °C). The cured hybrid resins display improved toughness. Typically, the critical stress intensity factor (K*_IC_*) and impact strength of OGCPS-EP-0.6-C are 2.54 MPa∙m^−1/2^ and 19.33 kJ∙m^−2^, respectively, which increased by 58.75% and 22.48% compared with the pristine epoxy resin, respectively. In addition, the glass transition temperature and flexural strength of the hybrid resins are basically unchanged.

## 1. Introduction

Aerospace technology, being a highly sophisticated and state-of-the-art field, serves as a significant emblem of advancements in modern science and technology. The realization of this technology is inseparable from the support of advanced materials. In the aerospace field, due to its extremely harsh operating environment, the material requirements of performance are more stringent. High-performance epoxy resins have found extensive applications as matrix resins in aerospace component composites owing to their good thermal stability [1], high glass transition temperature, excellent mechanical properties, and low curing shrinkage [2,3,4,5,6]. However, the high crosslinking density of the epoxy resins also results in low toughness, which significantly limits their usage in some situations. Therefore, enhancing the toughness of the epoxy resins has always been one of the key subjects for high-performance epoxy resins.

There are organic and inorganic toughening agents for toughening epoxy resins. Organic toughening agents include liquid rubbers [7,8], thermoplastic polymers [9,10,11], hyperbranched polymers [12,13], and liquid crystal polymers [14]. They have a good toughening effect, but usually at the expense of the mechanical and thermal properties of the epoxy resins. In addition, because the load amount of the agents is generally high, the viscosity of the modified epoxy resins will increase, which is not conducive to the processing of the resins. Inorganic toughening agents include graphene oxide [15,16,17,18], carbon nanotubes [19,20], nano-silica [21,22], and nano-clay [23]. They can maintain good mechanical properties while toughening epoxy resins. However, they are easy to aggregate in the resins because of the small size and large specific surface area of the nanoparticles, which form stress concentration points and reduce the performance of the resins.

Polyhedral oligomeric silsesquioxane (POSS), a kind of nano organic–inorganic hybrid molecule, is composed of a silicon and oxygen skeleton and has attracted extensive attention from researchers in recent years [24,25]. The general molecular formula of POSS can be abbreviated as (RSiO_1.5_)_n_ (where R stands for an organic group and n could be 8, 10, or 12). The center of POSS is a rigid inorganic Si-O-Si core and the exterior is connected to soft organic groups. POSS can be diversified through different chemical reactions, which can make POSS meet various application requirements. According to different external organic groups, POSSs can be divided into reactive POSSs and non-reactive POSSs. As a toughening agent for an epoxy resin, reactive POSSs can participate in the curing reaction of the epoxy resin through organic groups [26,27,28,29,30,31], and non-reactive POSSs can be uniformly dispersed in the resin by physical blending [32]. The molecular dispersion of reactive POSSs can be achieved through participation in the curing reaction of the epoxy resin, thereby enhancing the compatibility between POSSs and the epoxy resin.

Liu et al. [25] toughened an epoxy resin with POSS-grafted polyurethane (POSS-PU). The impact strength of the toughened epoxy resin increased by 115%, but the viscosity of the resin increased and the glass transition temperature decreased. Yang et al. [33] mixed octavinyl polyhedral oligomeric silsesquioxane (EPOSS)-modified vinyl resin with epoxy resin to form an interpenetrating network structure to toughen the epoxy resin. The impact strength of the resin increased by 139.6%, but the glass transition temperature decreased. Mishra et al. [28] incorporated trisilanol phenyl POSS and glycidyl POSS separately into an epoxy resin (EPON 862). Obviously, the trisilanol phenyl POSS is a non-reactive POSS and the glycidyl POSS is a reactive POSS. The toughness of the incorporated epoxy resins is increased by 30% and 130%, respectively. The toughening efficiency of the reactive POSS is much higher than that of the non-reactive POSS. Han et al. [26] modified an epoxy resin with octa-glycidyl POSS (OG-POSS) as a toughening agent. OG-POSS participated in the curing reaction and was evenly dispersed in the epoxy resin. The modified resin not only maintained the original glass transition temperature, flexural strength, and thermal stability but also increased the impact strength by 56%.

In this study, in view of the nano-sized hollow structure of polyhedral oligomeric silsesquioxanes and their good compatibility with a resin, three reactive polyhedral oligomeric silsesquioxanes with a thiol group (OMPPS), a carboxyl group (OCOPS), and an epoxy group (OGCPS) were synthesized and incorporated into the blend of 3-(oxiran-2-ylmethoxy)-*N*,*N*-bis(oxiran-2-ylmethyl)aniline/4,4′-methylenebis(2-ethylaniline) (epoxy resin) to obtain OMPPS-EP-i, OCOPS-EP-j, and OGCPS-EP-k hybrid resins with good comprehensive properties. The effect of the structure and content of the three modifiers on the curing behavior of the hybrid resins, as well as the thermal and mechanical properties of the cured resins, was investigated and compared.

## 2. Experimental

### 2.1. Materials

(3-Mercaptopropyl) trimethoxysilane (analytical purity), 10-undecylenoic acid (analytical purity), and 2,2′-azobis(2-methylpropionitrile) (AIBN) (analytical purity) were purchased from Shanghai Titan Technology Co., Ltd. 4-Hydroxybenzaldehyde (analytical purity) was bought from Jiangsu Anhuai Chemical Technology Co., Ltd. (Nantong, China). Methyl triphenyl phosphonium bromide (analytical purity) was supplied by Shanghai Zhonghe Chemical Technology Co., Ltd. (Shanghai, China). Potassium tert-butanol (analytical purity) was purchased from Shanghai XINBO Chemical Technology Co., Ltd. (Shanghai, China). Sodium hydride (analytical purity), epichlorohydrin (analytical purity), tetrabutylammonium bromide (analytical purity), and sodium hydroxide (analytical purity) were bought from Shanghai Boer Chemical Technology Co., Ltd. (Shanghai, China). Hydrochloric acid was bought from Sinopharm Chemical Reagent Co., Ltd. (Shanghai, China). Epoxy resin AFG-90MH (industrial grade) was supplied by Shanghai Huayi Resin Co., Ltd. (Shanghai, China). 4,4″-Methylenebis(2-ethylbenzenamine) (MOEA) (analytical purity) was purchased from Shanghai Bide Pharmaceutical technology Co., Ltd. (Shanghai, China). The release agent (909A 45) was supplied by Dongguan Jiadan lubricating oil Co., Ltd. (Dongguan, China). All solvents (analytical purity) were purchased from Shanghai Titan Technology Co., Ltd. (Shanghai, China).

### 2.2. Synthesis of Octa (Mercaptopropyl) Polyhedral Oligomeric Silsesquioxane (OMPPS)

**Scheme 1:**
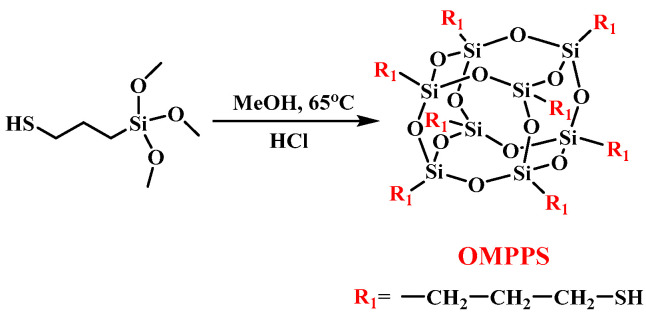
Synthetic route of OMPPS.

The synthetic route of OMPPS is shown in Scheme 1. First, 20 g of (3-Mercaptopropyl) trimethoxysilane (0.1 mol) and 25 mL of concentrated hydrochloric acid (36%) were added to a 1 L three-necked flask which contained 500 mL of methanol and stirred at 65 °C for 24 h under a N_2_ atmosphere. At the end of the reaction, the precipitate at the bottom of the flask was collected, washed three times with methanol, and dissolved in dichloromethane. Then, the obtained solution was washed three times with deionized water and dried to remove the solvent, followed by vacuum drying in a vacuum oven at 40 °C for 8 h to obtain OMPPS with a yield of 75%.

### 2.3. Synthesis of Octa (Carboxyl Decyl Thiopropyl) Polyhedral Oligomeric Silsesquioxane (OCOPS)

**Scheme 2:**
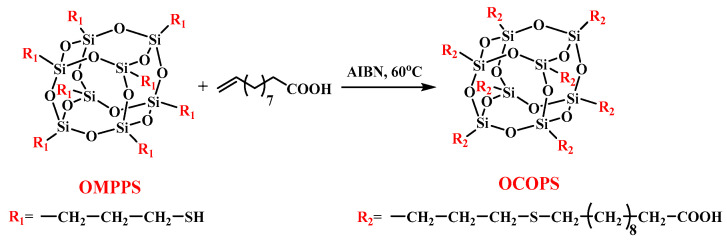
Synthetic route of OCOPS.

The synthetic route of OCOPS is shown in Scheme 2. First, 18.4 g of undecylenic acid (0.1 mol) and 1.0 g of 2,2′-azobis(2-methylpropionitrile) (AIBN) (6 mmol) were added to a 250 mL three-necked flask which contained 70 mL of dried tetrahydrofuran. The mixture in the flask was stirred until the solids were completely dissolved and heated up to 60 °C under N_2_. Then, a tetrahydrofuran solution (50 mL) containing 10.2 g (0.01 mol) OMPPS was added dropwise to the flask while stirring, and the addition time was controlled at 10~15 min. The reaction was stopped after maintaining at 60 °C for 6 h. After the reaction, the solution was concentrated and poured into 300 mL of methanol for sedimentation, and a white precipitate product was obtained. The product was placed in a vacuum oven and dried at 60 °C for 8 h to obtain OCOPS with a yield of 57%.

### 2.4. Synthesis of Octa (Glycidyl Phenyl Ethyl Thiopropyl) Polyhedral Oligomeric Silsesquioxane (OGCPS)

**Scheme 3:**
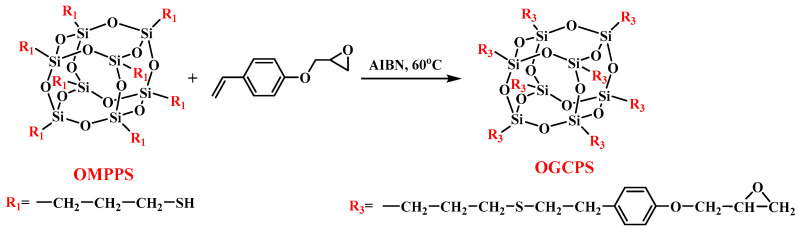
Synthetic route of OGCPS.

The synthetic route of OGCPS is shown in Scheme 3. 4-Vinyl phenyl glycidyl ether (DGHDS) was synthesized from 4-hydroxy styrene (HDS) and epichlorohydrin, which is shown in the supporting information.

First, 17.6 g of DGHDS (0.1 mol) and 1.0 g of 2,2′-azobis(2-methylpropionitrile) (AIBN) (6 mmol) were added to a 250 mL three-necked flask which contained 100 mL of dried tetrahydrofuran. The mixture was stirred until the solids were completely dissolved and heated up to 60 °C under N_2_. Then, a tetrahydrofuran solution (50 mL) containing 10.2 g (0.01 mol) OMPPS was added dropwise to the flask while stirring, and the addition time was controlled at 10~15 min. The reaction was stopped after maintaining at 60 °C for 6 h. After the reaction, the solution was concentrated and poured into 300 mL of methanol for sedimentation, and a light-yellow oily precipitate was obtained. After washing with methanol three times, the light-yellow oily precipitate was placed in a vacuum oven and dried at 60 °C for 8 h to obtain OMPPS with a yield of 52%.

### 2.5. Preparation of Hybrid Resins

**Figure 1:**
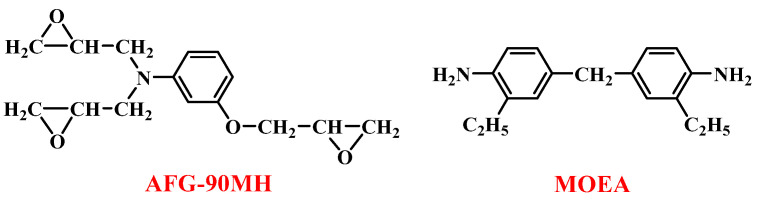
Structural formulas of AFG-90MH and MOEA.

The epoxy resin used in this study is 3-(oxiran-2-ylmethoxy)-*N*,*N*-bis(oxiran-2-ylmethyl)aniline (AFG-90MH), and the curing agent is 4,4′-methylenebis(2-ethylaniline) (MOEA). The structural formulas of AFG-90MH and MOEA are shown in Figure 1. The pristine epoxy resin (EP) used as the control group consisted of AFG-90MH and MOEA. The obtained reactive polyhedral oligomeric silsesquioxanes (OMPPS, OCOPS, and OGCPS) were separately mixed into the EP to obtain the hybrid resins. The molar ratio of active hydrogen to the epoxy group in the hybrid resins was controlled at 1:1.

#### 2.5.1. Preparation of OMPPS- or OGCPS-Modified Epoxy Hybrid Resins

AFG-90MH, MOEA, and OMPPS were dissolved in THF in a flask under stirring. After removing THF using a vacuum at 60 °C, the OMPPS-modified epoxy hybrid resins were obtained and named as OMPPS-EP-i (i = 0.3, 0.8, 1.4, 1.9, 2.8). The OGCPS-modified epoxy resins were made in the same way and named as OGCPS-EP-j (j = 0.1, 0.3, 0.6, 0.8, 1.2). The i and j are the molar percentage of OMPPS and OGCPS in the hybrid resins, respectively.

#### 2.5.2. Preparation of OCOPS-Modified Epoxy Hybrid Resins

First, OCOPS was dissolved in DMF in a flask, and the produced solution was mixed with AFG-90MH and prepolymerized at 110 °C for 0.5 h. Then, DMF was removed using a vacuum, and a homogeneous resin was obtained. Finally, MOEA was added to the resin to obtain the OCOPS-modified epoxy hybrid resins, named OCOPS-EP-k (k = 0.1, 0.3, 0.6, 0.8, 1.2, 1.6, 1.9), in which k is the molar percentage of OCOPS in the OCOPS-modified epoxy resins.

### 2.6. Preparation of Cured Hybrid Resins

The obtained hybrid resins were poured into the treated molds, respectively, and then the molds containing the resins were placed in a vacuum oven and evacuated at 90 °C for 0.5 h to remove the residual solvent. Afterward, the resins with molds were moved to an oven and heated to 110 °C, then kept at 110 °C for 2 h and 150 °C for another 2 h to obtain the cured resins. The obtained cured resins were abbreviated as EP-C, OMPPS-EP-i-C, OCOPS-EP-j-C, and OGCPS-EP-k-C, respectively.

### 2.7. Instruments and Measurements

The ^1^H NMR spectrum was obtained on the AVANCE 400 Ⅲ spectrometer (Bruker, Switzerland) at an operating frequency of 400 MHz with chloroform-D (CDCl_3_) or DMSO-*d6* as the deuterated solvent and tetramethylsilane as the internal standard. The FT-IR spectrum was obtained using a Nicolet iS10 infrared spectrometer (Thermo Scientific, Madison, WI, USA). Elemental analysis tests were conducted on the VARIO EL CUBE elemental analyzer (ELEMENTAR, Hanau, Germany). The relative molecular masses of POSSs were obtained by 4800 plus time-of-flight mass spectrometry (ABS, MAS Building, Singapore). The ^29^Si NMR spectrum was obtained on the Ascend 600 spectrometer at an operating frequency of 600 MHz with CDCl_3_ or DMSO-*d6* as the deuterated solvent and chromium acetylacetonate as the relaxation reagent. Differential scanning calorimetry (DSC) was run on a DSC214 (NETZSCH, Selb, Germany) instrument at the heating rate of 10 °C/min under nitrogen flow from 40 °C to 300 °C. Dynamic thermomechanical analysis (DMA) was performed on a DMA 1 mechanical analyzer (Mettler Toledo, Greifensee, Switzerland) by using the three-point bending mode at a heating rate of 5 °C/min with a frequency of 1 Hz under nitrogen flow. The flexural performance and fracture toughness of the cured resin were measured using a SANS CMT 4204 universal testing machine (Sansi Material Testing Co., Ltd., Shenzhen, China) at a speed of 2.00 mm/min and 1.5 mm/min, respectively, at room temperature with the three-point bending method. The impact performances of the cured resins were obtained by a CEAST 9050 cantilever beam impact testing machine (CEAST, Torino, Italy). The fracture surface of the cured resins was characterized by S-4800/S-3400 scanning electron microscopy (HITACHI, Tokyo, Japan).

## 3. Results and Discussion

### 3.1. The Structural Characterization of OMPPS, OCOPS, and OGCPS

**Figure 2:**
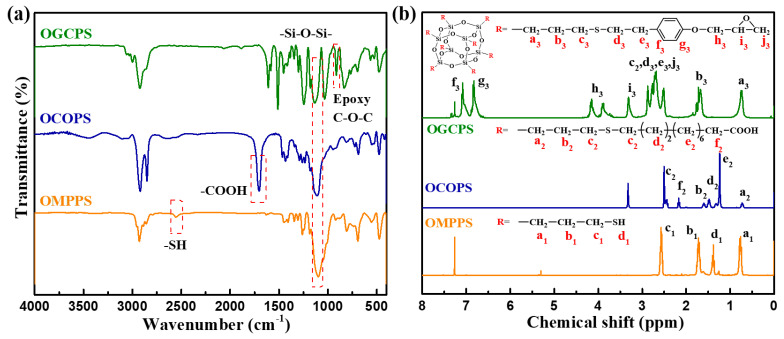
(**a**) FT-IR spectra of OMPPS, OCOPS, and OGCPS and (**b**) ^1^H NMR spectra of OMPPS, OCOPS, and OGCPS.

The structures of OMPPS, OCOPS, and OGCPS were characterized by FT-IR spectroscopy, and the spectra are shown in Figure 2a. The absorption peaks around 1100 cm^−1^ are the Si-O-Si stretching vibration peaks in OMPPS, OCOPS, and OGCPS. The peak at 2554 cm^−1^ corresponds to the -SH of OMPPS. The absorption peak of -SH disappears and the peak of -COOH at 1703 cm^−1^ for OCOPS appears, indicating that OCOPS has been successfully synthesized. In the FT-IR spectrum of OGCPS, the absorption peak of -SH also disappeared, and the absorption peak of the epoxy group appeared at 915 cm^−1^, indicating that the -SH at the eight corners of OMPPS has completely reacted with the vinyl group of DGHDS to form OGCPS. Figure 2b shows the ^1^H NMR spectra of OMPPS, OCOPS, and OGCPS. The solvent of the ^1^H NMR analysis for OMPPS and OGCPS is CDCl_3_ and the solvent for OCOPS is DMSO-*d6*. In the ^1^H NMR spectrum of OMPPS, the resonance peaks for the hydrogens of three methylene in the mercaptopropyl group are found at chemical shifts of 0.77 ppm(a_1_), 1.72 ppm(b_1_), and 2.57 ppm(c_1_), respectively, and the resonance peak of sulfhydryl hydrogen is found at chemical shift of 1.38 ppm(d_1_). The area ratio of the peaks for the hydrogens at a_1_, b_1_, c_1_, and d_1_ is 2.07:2.14:1.98:1.0. In the ^1^H NMR spectrum of OCOPS, the chemical shifts of the methylene hydrogens at a_2_, b_2_, and c_2_ move to the high field under the influence of the long-chain alkane group, compared to those of the hydrogens of OMPPS at a_1_, b_1_, and c_1_, which are 0.73 ppm, 1.60 ppm, and 2.50 ppm, respectively. The resonance peak of sulfhydryl hydrogen disappears, indicating that the sulfhydryl group has completely participated in the reaction, and the eight corners of POSS have become carboxyl-terminated groups. Under the influence of the thioether group, the chemical shifts of hydrogens for methylene-connected sulfur atoms are close, at about 2.50 ppm. The chemical shift of methylene hydrogens at d_2_ is influenced by the thioether group and moves to the lower field, at 1.47 ppm, while the chemical shift of methylene hydrogens at e_2_ is less affected and is 1.24 ppm. Due to the influence of the carboxyl group, the resonance peak of methylene hydrogens connected with the carboxyl group moves to the lower field and is 2.17 ppm. The peak area ratio is 2.00:2.05:4.07:3.98:12.14:2.0 for hydrogens at a_2_, b_2_, c_2_, d_2_, e_2_, and f_2_, respectively. In the ^1^H NMR spectrum of OGCPS, the resonance peaks at a_3_, b_3_, and c_3_ are slightly shifted to the lower field as compared to the hydrogens of OMPPS at a_1_, b_1_, and c_1_, with chemical shifts of 0.74 ppm, 1.68 ppm, and 2.53 ppm, respectively. The resonance peak of sulfhydryl hydrogen disappears, indicating that the sulfhydryl group has completely reacted, and the eight corners of POSS have become hydrocarbon chains with a terminal epoxy group. In addition, the chemical shifts of other hydrogen atoms in OGCPS can also correspond with each other in the spectrum. The peak area ratio of the hydrogens at a_3_, b_3_, (c_3_ + d_3_ + j_3_ + e_3_), i_3_, h_3_, g_3_, and f_3_ is 2.14:2.18:7.92:1.00:2.14:2.08:2.0.

**Figure 3:**
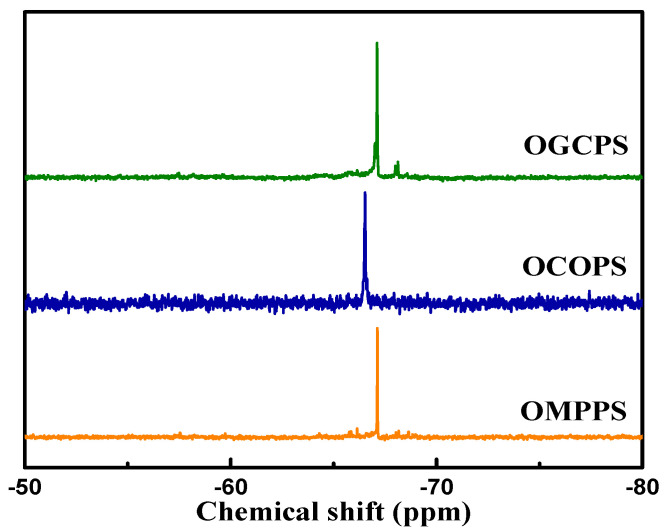
^29^Si NMR spectra of OMPPS, OCOPS, and OGCPS.

The ^29^Si NMR spectra of OMPPS, OCOPS, and OGCPS are shown in Figure 3. The solvent of the ^29^Si NMR analysis of OMPPS and OGCPS is CDCl_3_ and the solvent for OCOPS is DMSO-*d6*. The ^29^Si NMR spectra of OMPPS, OCOPS, and OGCPS show a large single peak, indicating that the purity is high. In addition, the relative molecular masses of OMPPS, OCOPS, and OGCPS were determined by using MALDI-TOF-MS, and the results are shown in Figure S2. The analysis results are as follows: OMPPS (product + Na^+^): 1039.20 Da (calculated: 1039.14), OCOPS (product + Na^+^): 2512.33 Da (calculated: 2512.33), and OGCPS (product + Na^+^): 2447.30 Da (calculated: 2447.27). These results show that the molecular weights of OMPPS, OCOPS, and OGCPS are 1016, 2489, and 2424, respectively. The above test results demonstrate that OMPPS, OCOPS, and OGCPS were successfully synthesized.

The solubility of OMPPS, OCOPS, and OGCPS has an influence on the subsequent application. The dissolution behaviors of OMPPS, OCOPS, and OGCPS in common organic solvents are listed in Table 1. OMPPS and OGCPS have good solubility and can be dissolved in dichloromethane, tetrahydrofuran, ethyl acetate, *N*,*N*-dimethylformamide, and dimethyl sulfoxide. However, OCOPS has poor solubility and can be dissolved in tetrahydrofuran, *N*,*N*-dimethylformamide, and dimethyl sulfoxide.

### 3.2. The Processability of Hybrid Resins

**Figure 4:**
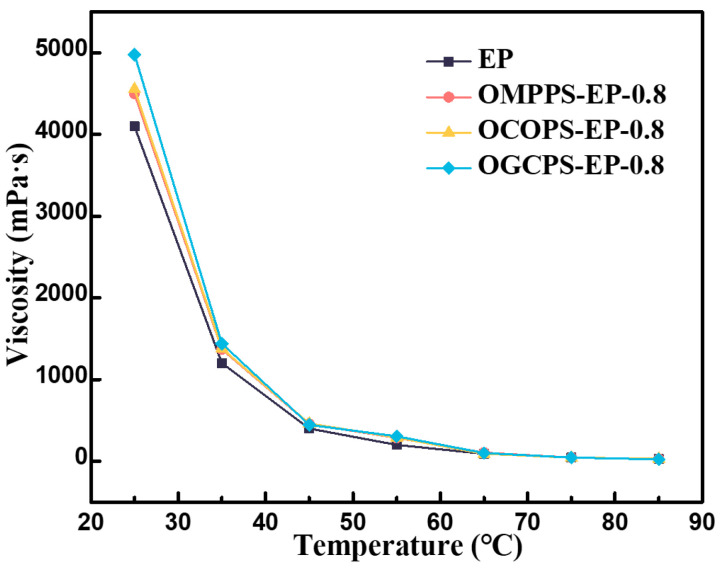
Viscosity of pristine epoxy resin, OMPPS-EP-0.8, OCOPS-EP-0.8, and OGCPS-EP-0.8.

Viscosity has an important effect on the processability of the epoxy resin. Figure 4 shows the viscosity of different resins as a function of the temperature. Obviously, the addition of OMPPS, OCOPS, and OGCPS has no significant effect on the viscosity of the epoxy resin. The viscosity of hybrid resins is lower than 500 mPa∙s at 45~85 °C, which is suitable for the liquid molding (RTM) process of composite materials.

### 3.3. Thermal Curing Behaviors of Hybrid Resins

**Figure 5:**
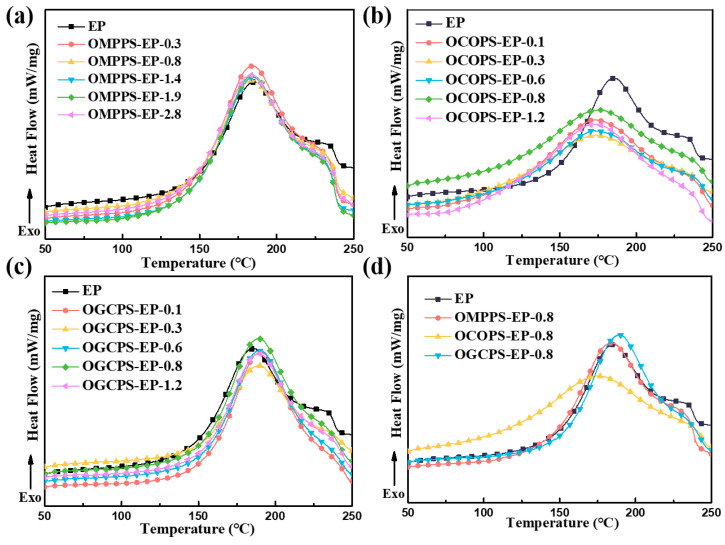
DSC curves of the OMPPS-EP-i (**a**), OCOPS-EP-j (**b**), and OGCPS-EP-k (**c**) hybrid resins. (**d**) The pristine epoxy resin and hybrid resins with the same load amount of modifier.

In order to investigate the curing behavior of the hybrid resins, a differential scanning calorimeter (DSC) was used. Figure 5a–c shows the DSC curves of the OMPPS-EP-i, OCOPS-EP-j, and OGCPS-EP-k hybrid resins, and the detailed analysis data are listed in Table S1. It can be seen that the addition of OMPPS and OGCPS has no effect on the curing behaviors of the epoxy resin, but the addition of OCOPS can slightly reduce the curing temperature. This may be due to the fact that the carboxyl groups in OCOPS have a higher reactivity with epoxy resin than active groups in OMPPS and OGCPS. In order to compare the effects of different modifiers on the thermal curing behavior of the epoxy resin, we selected three hybrid resins with a modifier addition amount of 0.8 mol% for comparison. Figure 5d shows the DSC curves of the epoxy resin and hybrid resins with a 0.8 mol% load of the modifier. It can be seen that under conditions where the same molar amount is added, OCOPS is beneficial in promoting the thermal cross-linking reaction of epoxy resin.

**Figure 6:**
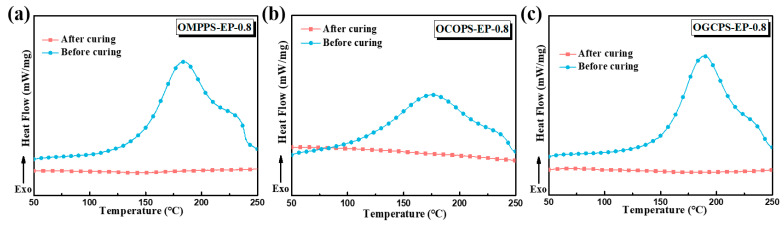
DSC curves of OMPPS-EP-0.8 (**a**), OCOPS-EP-0.8 (**b**), and OGCPS-EP-0.8 (**c**) before and after curing.

The *T*_i_ (initial curing temperature) and *T*_p_ (top curing peak temperature) of the modified resins usually vary with the heating rate. The higher the heating rate, the higher the *T*_i_ and *T*_p_. We conducted DSC tests for the pristine epoxy resin at various heating rates. The *T*_i0_ and *T*_p0_ at the heating rate of zero are obtained through the linear extrapolation of the *T*_i_ and *T*_p_ with the heating rate (Figure S4), and are 110 °C and 155 °C, respectively. Therefore, 110 °C/2 h + 150 °C/2 h was selected as the curing process of the epoxy resin. Figure 6 shows the DSC curves of OMPPS-EP-0.8, OCOPS-EP-0.8, and OGCPS-EP-0.8 before and after curing. There is no obvious exothermic peak in the cured epoxy resins, indicating that the epoxy resins have been completely cured.

**Figure 7:**
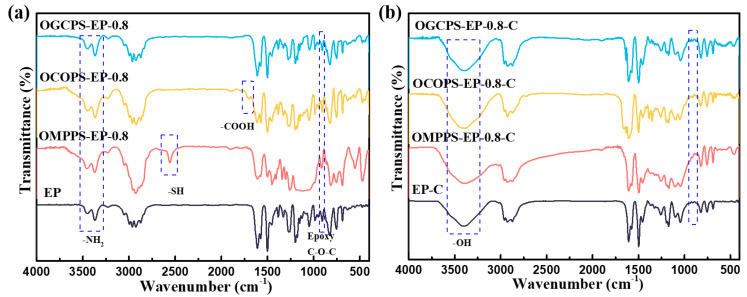
FT-IR spectra of the hybrid resins with 0.8 mol% modifier content before (**a**) and after (**b**) curing.

FT-IR spectroscopy was adopted to investigate the structural changes in the hybrid resins during the curing process. The FT-IR spectra of the hybrid resins with 0.8 mol% modifier content before and after curing are shown in Figure 7. As shown in Figure 7a, the absorption peaks at 3400~3500 cm^−1^ are assigned to the stretching vibration peak of the amino group in the curing agent (MOEA). The absorption bands at 2554 cm^−1^ and 1703 cm^−1^ are attributed to the stretching vibration of -SH and -COOH, respectively, indicating that the modifiers have been introduced into the epoxy resin. After curing, the absorption peaks of -NH_2_ for the curing agent and the absorption peaks of the epoxy group (915 cm^−1^) disappeared, and a new absorption peak of -OH appeared, indicating that the amino group and epoxy group have completely reacted. In addition, the absorption peak of -SH in OMPPS-EP-0.8 and the absorption peak of -COOH in OCOPS-EP-0.8 disappeared after curing, indicating that OMPPS and OCOPS have participated in the curing reaction of the epoxy resins.

### 3.4. Mechanical Properties of Cured Hybrid Resins

#### 3.4.1. Flexural and Impact Properties of Cured Hybrid Resins

**Figure 8:**
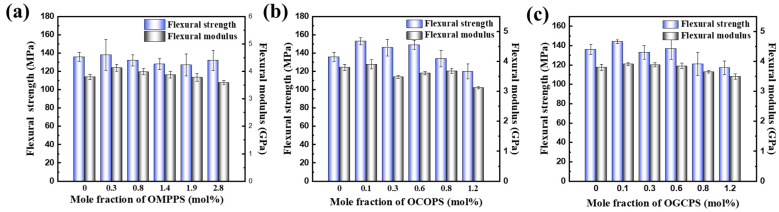
Flexural properties of OMPPS-EP-i-C (**a**), OCOPS-EP-j-C (**b**), and OGCPS-EP-k-C (**c**).

The toughness and strength of resins have a significant influence on the performance of terminal fiber reinforced composites. The flexural properties of the cured hybrid resins were tested and the results are shown in Figure 8. When the amount of modifier added is small, the flexural properties of the cured hybrid resins increase slightly and then reach a peak value. However, as the amount of modifier added increases, the flexural properties begin to decline slightly. The reason for this phenomenon may be that at low loading, the rigid inorganic nano-scale Si-O-Si core is uniformly dispersed, which can disperse the stresses concentrated on the crack tip and thus increase the flexural properties of the cured hybrid resins. However, with the continuous addition of the POSS modifiers, POSS molecules self-assemble and aggregate to form the so-called “hard-core soft-shell particles”, which play a plasticizing role, resulting in a decline in the flexural properties of cured hybrid resins [28].

As shown in Figure 8, the flexural strength of OMPPS-EP-0.3-C reaches 138 MPa, compared with the flexural strength of 136 MPa for EP-C. Compared with OMPPS, OCOPS has longer and more flexible alkyl chain segments, the distance between the molecular chains is increased, and the free volume fraction in the cured resin is increased. Thus, the flexural strength of OCOPS-EP-0.6-C increases to 149 MPa, which is 10% higher than that of EP-C. As for OGCPS-EP-0.6-C with the same modifier addition ratio, the flexural strength of OGCPS-EP-0.6-C reaches 137 MPa, which is close to that of the pristine resin. This is mainly due to OGCPS’s molecular structure, which contains rigid benzene rings, increasing the rigidity of the cured resins (flexural modulus: OGCPS-EP-0.6-C > OCOPS-EP-0.6-C) and restricting the movement of the molecular chain. In summary, these results show that OMPPS, OCOPS, and OGCPS can effectively maintain or improve the flexural properties of epoxy resins at a certain dosage.

**Figure 9:**
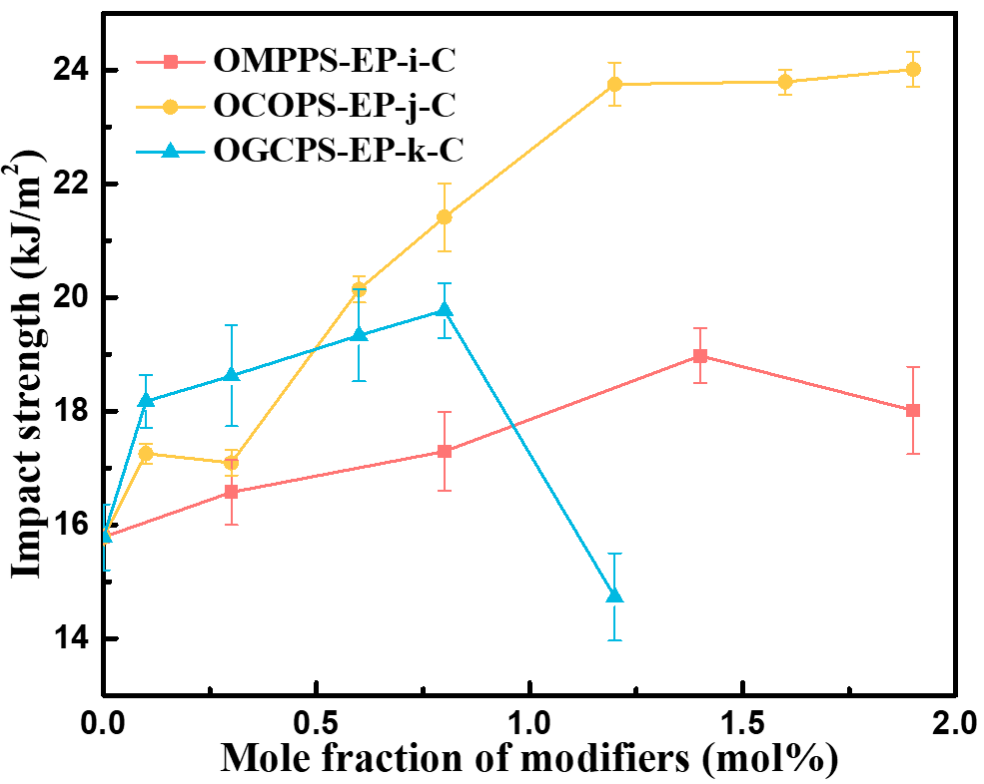
Impact strength of OMPPS-EP-i-C, OCOPS-EP-j-C, and OGCPS-EP-k-C.

Impact performance tests were conducted on the cured resins, and the results are shown in Figure 9. It can be seen that the impact strength of OMPPS-EP-i-C and OGCPS-EP-j-C increases first and then decreases with an increase in the amount of modifier added, while the impact strength of OCOPS-EP-k-C increases with an increase in the amount of OCOPS added.

OMPPS, OCOPS, and OGCPS contain functional groups that can participate in the curing reactions of the epoxy resin, which can cause them to become better dispersed in the resin. POSSs, with rigid -Si-O-Si- cage-like structures, are uniformly distributed in the epoxy networks. When the cured epoxy structures are subjected to impact, POSSs play a dual role. On the one hand, they are able to absorb some energy as stress concentration points and thereby facilitate the dispersion of the stress. On the other hand, they also prevent the formation and propagation of micro-cracks, while forming shear bands within the resins to absorb another portion of the impact energy [26,28,34]. Organic soft segments in POSSs can also increase the flexibility of the polymer chain [35,36,37] and thus increase the impact strength of the epoxy resins. However, the impact strength decreases with the further loading of the amount of POSSs. The decline is possibly related to the assembling of POSSs.

**Figure 10:**
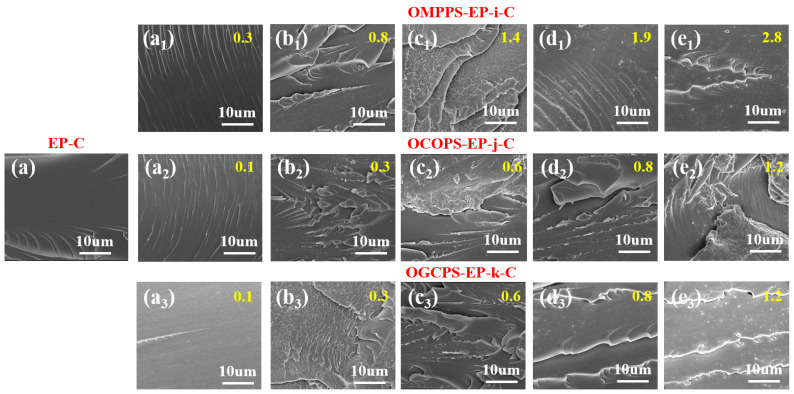
SEM images of the fracture surfaces of EP-C (**a**), OMPPS-EP-i-C (**a_1_**–**e_1_**), OCOPS-EP-j-C (**a_2_**–**e_2_**), and OGCPS-EP-k-C (**a_3_**–**e_3_**).

In order to further analyze the relationship between the impact strength of the hybrid resins and the type and amount of modifier added, the micromorphology of the cured resins was investigated. The fracture surfaces of cured resins were observed by scanning electron microscopy (SEM), and the results are shown in Figure 10. As can be seen from Figure 10, EP-C presents a smooth fracture surface, indicating that the break of the resin is a brittle fracture. With the addition of OMPPS, OCOPS, and OGCPS, the fracture surfaces of the cured resins become rough, indicating that the fracture mode changes from brittleness to toughness [25,38,39]. For OMPPS-modified epoxy resins and OGCPS-modified epoxy resins, the roughness of the fracture surfaces first increases and then decreases with increases in POSS load amount. For OCOPS-modified epoxy resins, the roughness of the fracture surfaces increases with the increase in POSS load amount. The results demonstrate the change in impact strength.

#### 3.4.2. Fracture Toughness of Cured Hybrid Resins

**Figure 11:**
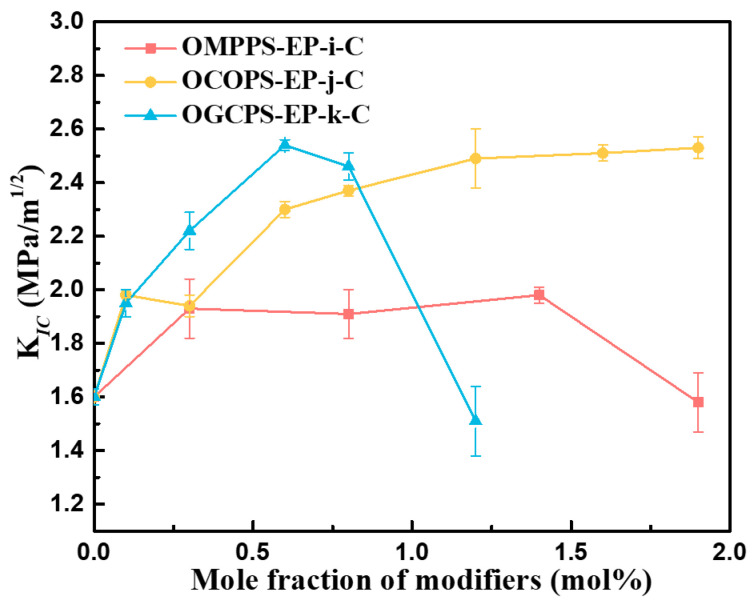
K*_IC_* of OMPPS-EP-i-C, OCOPS-EP-j-C, and OGCPS-EP-k-C.

Fracture toughness refers to the ability of a material to prevent the unstable propagation of macro cracks, which can generally be represented by the critical stress intensity factor (K*_IC_*). The larger the K*_IC_*, the better the fracture toughness of the material. Figure 11 shows the K*_IC_* of OMPPS-EP-i-C, OCOPS-EP-j-C, and OGCPS-EP-k-C.

As shown in Figure 11, the K*_IC_* of OMPPS-EP-i-C and OGCPS-EP-k-C increases first and then decreases with the increase in the amount of modifier added, but the toughening effect of OGCPS is much better than that of OMPPS. The K*_IC_* of the pristine epoxy resin is 1.60 MPa∙m^−1/2^. When the addition amounts of OMPPS and OGCPS were 1.4 mol% and 0.6 mol%, respectively, the K*_IC_* of the cured resin reached the maximum, which was 1.98 MPa∙m^−1/2^ and 2.54 MPa∙m^−1/2^. Compared with the K*_IC_* of EP-C, it increased by 23.75% and 58.75%. The fracture toughness of OCOPS-modified epoxy resin increases with the increase in OCOPS addition. When the addition amount is 1.9 mol%, the K*_IC_* of OCOPS-EP-1.9-C is 2.53 MPa∙m^−1/2^, which is increased by 58.13% and close to the toughness of the OGCPS-EP-0.6-C resin.

There are two factors that affect the fracture toughness of the hybrid resins. One is the load capacity of OMPPS, OCOPS, and OGCPC in the epoxy resin, and the other is the different organic chain segments and functional groups in OMPPS, OCOPS, and OGCPC. When OMPPS or OGCPS is added to the epoxy resin, the fracture toughness of the hybrid resins first increases and then decreases, and there is a maximum value. After reaching the maximum value, further addition of OMPPS or OGCPS leads to a decrease in fracture toughness. This probably results from the aggregation of modifiers at a high load amount, which form stress concentration points within the resin when the resin is stressed [29,40]. The aggregation of the modifiers was observed by SEM, and the results are shown in Figure S5. Unlike OMPPS and OGCPS, the fracture toughness of the OCOPS-modified epoxy resins increases with the increase in OCOPS load amount. This may probably result from the long and flexible aliphatic chains at the eight corners of OCOPS.

In addition to the load capacity of OMPPS, OCOPS, and OGCPC, the difference in the organic segments of OMPPS, OCOPS, and OGCPC also leads to different toughening effects on the epoxy resin. OCOPS and OGCPS exhibit superior performance in enhancing the toughness of epoxy resins compared to OMPPS. This is primarily attributed to the longer molecular chain segments of OCOPS and OGCPS than those of OMPPS. These long flexible chain segments allow them to easily move and absorb more energy when the resins are subjected to stress. In addition, OGCPS exhibits better toughening effects on the epoxy resins compared to OCOPS before OGCPS agglomerates in the resin. This is possibly due to the presence of benzene rings in the organic chain segments and the glycidyl groups of OGCPS [36].

### 3.5. Thermal Properties of Cured Hybrid Resins

**Figure 12:**
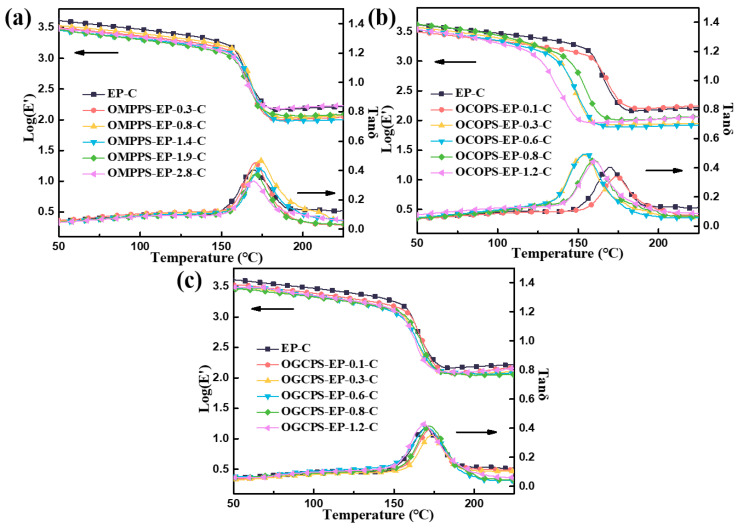
DMA curves of EP-C, OMPPS-EP-i-C (**a**), OCOPS-EP-j-C (**b**), and OGCPS-EP-k-C (**c**) in nitrogen.

Dynamic mechanical analysis (DMA) was used to analyze the thermal mechanical properties of the cured resins, and the results are shown in Figure 12. As can be seen from Figure 12, OMPPS-EP-i-C, OCOPS-EP-j-C, and OGCPS-EP-k-C have a lower storage modulus (E’) than EP-C. All three modifiers have a caged hollow structure and flexible organic chain segments on the outside. Introducing them into the epoxy resin will lead to an increase in the free volume of the hybrid resin, causing a decrease in the storage modulus. The crosslinking density of the cured resins can be calculated using the data from the DMA curves, and the results are shown in Table S2. The results show the crosslinking density of the cured hybrid resins is lower than that of the cured pristine epoxy resin, which indicates the toughness contribution. The presence of a single peak in all tanδ curves indicates good compatibility between OMPPS, OCOPS, OGCPC, and the epoxy resin. The temperature corresponding to the peak is the glass transition temperature (*T*_g_) of the cured resin. The *T*_g_ values of all epoxy resins are listed in Table 2. The introduction of OMPPS and OGCPS had no significant effect on the *T*_g_ of the cured hybrid resin. This is because OMPPS and OGCPS both have rigid internal Si-O-Si structures and external flexible organic chain segments, and the effects of the two on the *T*_g_ offset each other. However, the *T*_g_ of OCOPS-EP-j-C had a significant decreasing trend with increase in OCOPS addition. This may be because the external organic chain segments of OCOPS are longer or more flexible than those of OMPPS and OGCPS. At a lower loading, the effect of the rigid Si-O-Si structure on the *T*_g_ and the effect of flexible chain segments on the *T*_g_ offset each other, so the *T*_g_ of OCOPS-EP-0.1-C is close to that of EP-C. However, at higher addition amounts, due to the longer molecular chain of OCOPS, OCOPS has a stronger plasticizing effect, which exceeds the effect of the rigid Si-O-Si structure on the *T*_g_ of the modified epoxy resin [41]. Therefore, the *T*_g_ of OCOPS-EP-j-C decreases.

**Figure 13:**
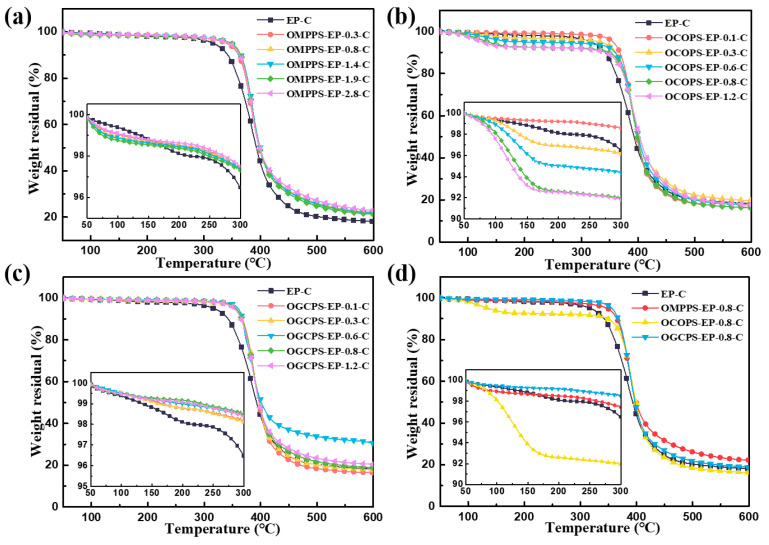
TGA curves of EP-C, OMPPS-EP-i-C (**a**), OCOPS-EP-j-C (**b**), and OGCPS-EP-k-C (**c**), and modifier-EP-0.8-C (**d**) in nitrogen.

Thermogravimetric (TGA) analysis was used to determine the thermal stability of the resins, and the results are shown in Figure 13. The *T*_d5_ values of the cured hybrid resins are listed in Table 2. As shown in Table 2, we can see that the introduction of OMPPS and OGCPS can improve the thermal stability of the cured hybrid resins. Due to the short arms of OMPPS, most of which are composed of high energy Si-O and Si-C bonds [42,43], this is the key to the improvement of the thermal stability of OMPPS-EP-i-C. In contrast, OGCPS has a longer molecular chain segment than OMPPS, but the molecular chain of OGCPS contains a benzene ring structure, which also ensures that OGCPS-EP-k-C has high thermal stability [44]. However, for OCOPS-EP-j-C, the thermal stability is much lower than that of EP-C. There may be two reasons. Firstly, the presence of long flexible chains in the arms of OCOPS enhances the flexibility of the cross-linked structure when incorporated into the epoxy resin, subsequently compromising the thermal stability of the epoxy resin. Secondly, the reactive groups of OCOPS are carboxyl groups, and the ester groups formed during the curing reaction with epoxy groups may lead to a further reduction in the thermal stability of OCOPS-EP-j-C.

## 4. Conclusions

Three reactive polyhedral oligomeric silsesquioxanes with a thiol group (OMPPS), a carboxyl group (OCOPS), and an epoxy group (OGCPS) are utilized to modify 3-(oxiran-2-ylmethoxy)-*N*,*N*-bis(oxiran-2-ylmethyl)aniline/4,4′-methylenebis(2-ethylaniline) (epoxy resin) and obtain hybrid resins with good thermal and mechanical properties. The hybrid resins display low viscosity in the temperature range of 45~85 °C and a low curing temperature (110 °C), which is beneficial for the liquid molding of the resin composites. The addition of OMPPS, OCOPS, and OGCPS greatly improves the toughness of the epoxy resin without sacrificing the mechanical and thermal properties of the epoxy resin. The critical stress intensity factor (K*_IC_*), impact strength, and *T*_d5_ of OGCPS-EP-0.6-C reach 2.54 MPa∙m^−1/2^, 19.33 kJ/m^2^, and 357 °C, showing increases of 58.75%, 22.48%, and 12.62%, respectively, compared to EP-C. The toughness of cured hybrid resins first increases and then decreases with the increase in the addition amount of OMPPS or OGCPS, reaching the maximum value when the addition amount is 1.4 mol% and 0.6 mol%, respectively. The toughness of OCOPS-EP-j-C increases with the increase in OCOPS addition. OCOPS, with long and flexible chain arms, exhibits a better toughening effect. This study provides a strategy for the design of polyhedral oligomeric silsesquioxane toughening agents and application in an epoxy resin, demonstrating an effective toughening method for the resin matrices of advanced composites.

## Figures and Tables

**Table 1:** Solubility of OMPPS, OCOPS, and OGCPS.

Solvent	Solubility		
	OMPPS	OCOPS	OGCPS
Methanol	−	−	−
Ethanol	−	−	−
Tetrahydrofuran	+	+	+
Ethyl acetate	+	−	+
Dimethylformamide	+	+	+
Dimethyl sulfoxide	+	+	+
Dichloromethane	+	−	+
Petroleum ether	−	−	−
Chloroform	+	−	+

+ soluble, − insoluble.

**Table 2:** *T*_g_s and *T*_d5_s of EP-C, OMPPS-EP-i-C, OCOPS-EP-j-C, and OGCPS-EP-k-C.

Sample	*T*_g_ (°C)	*T*_d5_ (°C)
EP-C	169	317
OMPPS-EP-0.3-C	170	341
OMPPS-EP-0.8-C	173	347
OMPPS-EP-1.4-C	173	348
OMPPS-EP-1.9-C	171	349
OMPPS-EP-2.8-C	170	345
OCOPS-EP-0.1-C	174	356
OCOPS-EP-0.3-C	156	333
OCOPS-EP-0.6-C	155	204
OCOPS-EP-0.8-C	157	136
OCOPS-EP-1.2-C	158	125
OGCPS-EP-0.1-C	166	355
OGCPS-EP-0.3-C	168	356
OGCPS-EP-0.6-C	167	357
OGCPS-EP-0.8-C	167	356
OGCPS-EP-1.2-C	170	353

## Data Availability

Data are contained within the article.

## Supplementary Materials

The following supporting information can be downloaded at: https://www.mdpi.com/article/10.3390/polym16131877/s1, Scheme S1: Synthetic route of HDS; Scheme S2: Synthetic route of DGHDS; Figure S1: (a) FT-IR spectra of HDS and DGHDS and (b) ^1^H NMR spectra of HDS and DGHDS; Figure S2. MALDT-TOF-MS spectra of OMPPS, OCOPS, and OGCPS; Figure S3. The sample diagram of four mechanical properties tests; Figure S4: Linear fitting of *T*_i_ and *T*_p_ vs. heating rate for the pristine resin; Figure S5: SEM images of the fracture surface of OMPPS-EP-0.8-C (a_1_), OMPPS-EP-1.9-C (a_2_), OGCPS-EP-0.6-C (b_1_), and OGCPS-EP-1.2 (b_2_); Table S1: DSC analysis data of OMPPS-EP-i, OCOPS-EP-j, and OGCPS-EP-k hybrid resins; Table S2: The cross-linking density of cured epoxy resins. Reference [45] is cited in the supplementary materials.
