# Supplementary material for: Novel Reactive Polyhedral Oligomeric Silsesquioxane-Reinforced and Toughened Epoxy Resins for Advanced Composites
Source: Polymers (Basel). 2024 Jul 1;16(13):1877. doi: 10.3390/polym16131877 (PMC11243849; doi:10.3390/polym16131877)
Supplement: Supplementary file 1 [file polymers-16-01877-s001.zip › polymers-2999985-supplementary.pdf]

Supporting information

Novel POSS-reinforced and toughened epoxy resins for advanced composites

Weibo Liu [1], Caiyun Wang [1], Yu Feng [1], Yongfeng Chen [1], Liqiang Wan [1], Farong Huang [1,*], Zuozhen Liu [2], Jianhua Qian [2] and Weiping Liu [3]

[1] Key Laboratory for Specially Functional Polymeric Materials and Related Technology of (Ministry of Education), School of Materials Science and Engineering, East China University of Science and Technology, Shanghai 200237, China; y82210301@mail.ecust.edu.cn (W.L.); y10210074@mail.ecust.edu.cn (C.W.); fengyu10161366@163.com (Y.F.); 13918350379@163.com (Y.C.); wanliqiang@163.com (L.W.)

[2] HuaChang Polymers Co., Ltd., East China University of Science and Technology, Shanghai 200241, China; lzz@ecust.edu.cn (Z.L.); qjh@hchp.com.cn (J.Q.)

[3] Manufacturing Center of Composite Materials for Commercial Aircraft, Shanghai Aircraft Manufacturing Co., Ltd., COMAC, Shanghai 201324, China; liuweiping@comac.cc

* Correspondence: fhuanglab@ecust.edu.cn

1. Syntheses and characterization of the intermediates for POSS

1.1. Synthesis of 4-hydroxystyrene (HDS)

A 500 ml three-necked flask was subjected to anhydrous and anaerobic treatment. Methyl triphenyl phosphorus bromide (Ph_3PCH_3Br) 64.4 g (0.18 mol) and dry tetrahydrofuran 300 ml were added to the flask, and white suspension was formed by stirring vigorously under N_2 atmosphere for 5 min. Then potassium tert-butyl alcohol (KtBu) 36.8 g (0.32 mol) and sodium hydride (NaH) 2 g (0.8 mol) were added to the three-necked flask, and the homogenization was continued by stirring. 4-Hydroxybenzaldehyde 20 g (0.16 mol) was dissolved in 100 ml dry tetrahydrofuran. Then the produced solution was slowly added into the three-necked flask, and the

dropping time was controlled for 20~30 min. The reaction was stopped after maintaining constant temperature for 6 h. At the end of the reaction, 80 ml deionized water and 30 ml 10% dilute hydrochloric acid were added to the three-necked flask to adjust the pH of the mixture to 3~6. After standing, the solution in the three-necked flask was stratified. The organic phase was separated and washed three times with deionized water, and the solvent was removed by drying to obtain a crude product. The crude product was purified by column chromatography and HDS was obtained with the yield of 87%.

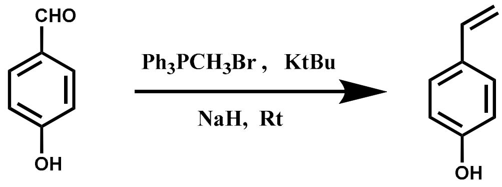

Scheme S1. Synthetic route of HDS

1.2. Synthesis of 4-vinyl phenyl glycidyl ether (DGHDS)

4-Hydroxystyrene (HDS) 12.0 g (0.1 mol), epiglorohydrin 50.0 ml and tetrabutylammonium bromide (TBAB) 1.61 g (5 mmol) were added to a 100 ml three-necked flask and stirred at 100 °C under N_2 atmosphere for 3 h. Then the reaction system was cooled to 40 °C. Thereafter, 40 ml 20wt% sodium hydroxide solution was added in the flask, and stirring was continued for 3 h at 40 °C. At the end of the reaction, the organic phase was separated, washed three times with deionized water, dried to obtain a crude product. The crude product was purified by column chromatography and DGHDS was obtained with the yield of 88%.

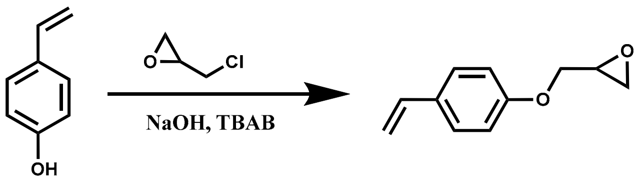

Scheme S2. Synthetic route of DGHDS

1.3. The structural characterization of HDS and DGHDS

As can be seen in Figure S1 (a), the peaks at 1605 cm^{-1} and 1509 cm^{-1} correspond to the absorption of benzene ring. There is a strong hydroxyl absorption peak at 3350 cm^{-1} in the FT-IR spectra of HDS, and an epoxy group absorption peak at 914 cm^{-1} in the FT-IR spectra of DGHDS, indicating that HDS successfully reacted with epichlorohydrin, and the phenol hydroxyl group was successfully changed into phenyl glycidyl ether. The structure characterization of HDS and DGHDS were further conducted by ^{1}H NMR analysis, as shown in Figure S1 (b). The solvents of the ^{1}H NMR analysis for HDS and DGHDS were DMSO-*d6* and $CDCl_3$, respectively. For HDS, the resonance peak of hydroxyl hydrogen at chemical shift 9.51 ppm (e_1), the resonance peaks of four hydrogens on the benzene ring at chemical shift 7.27 ppm (c_1) and 6.74 ppm (d_1), and the resonance peaks of vinyl hydrogen (=CH—) near the benzene ring at chemical shift 6.61 ppm (b_1). Chemical shifts of 5.56 ppm (a_1) and 5.02 ppm (a_1) are resonance peaks of other vinyl hydrogens (CH_2=) on the benzene ring, respectively. The peak area ratio is 2.02:1.00:2.01:2.03:0.97 for hydrogens at a_1, b_1, c_1, d_1 and e_1, which is close to 2:1:2:2:1 for the chemical structure of HDS. For DGHDS, the chemical shifts of the hydrogen on the benzene ring and the hydrogen on the vinyl group are 7.34 ppm (c), 6.87 ppm (d), 6.66 ppm (b), 5.59 ppm (a), 5.14 ppm

(a), respectively. Chemical shifts of 4.19 ppm (e) and 3.94 ppm (e) are the resonance peaks of phenoxy-linked methylene hydrogen, and chemical shifts of 3.33 ppm (f), 2.89 ppm (g) and 2.74 ppm (g) are the resonance peaks of methylene hydrogen in the epoxide group. The peak area ratio is 2.03:1.00:2.01:2.01:2.05:1.04:2.02 for hydrogens at a, b, c, d, e, f and g, which is close to 2:1:2:2:2:1:2 for the chemical structure of DGHDS.

EI-MS and elemental analysis were used to further determine the structure of HDS and DGHDS, and the results show HDS (m/z: 120.07; found: C: 80.01%; H: 6.70%; O: 13.29%, calc: C: 79.9%; H: 6.71%; O: 13.32%), DGHDS (m/z: 176.02; C: 74.98%; H: 6.88%; O: 18.14%, calc: C: 74.98%; H: 6.86%; O: 18.16%).

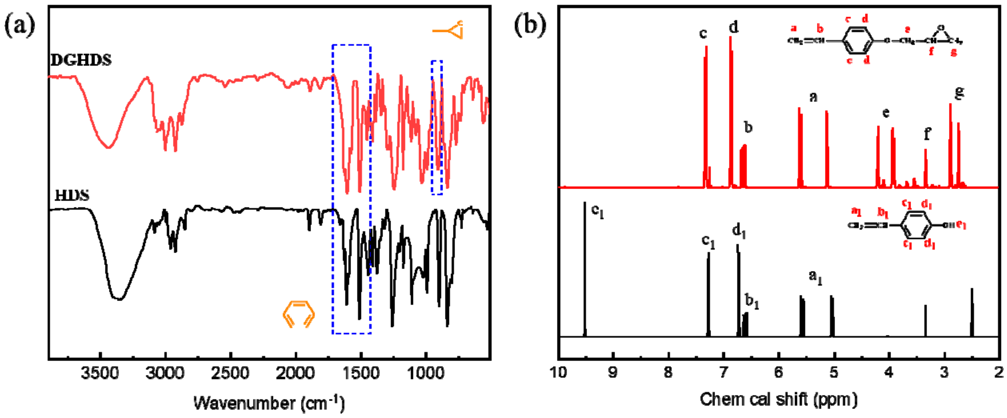

Figure S1. (a) FT-IR spectra of HDS and DGHDS, and (b) ^{1}H NMR spectra of HDS and DGHDS

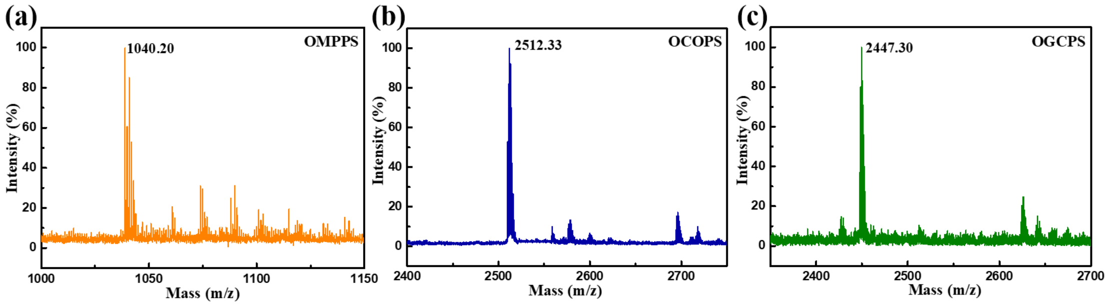

Figure S2. MALDT-TOF-MS spectra of OMPPS, OCOPS and OGCPS

2. The curing character of hybrid resins

Figure S2 is a sample diagram of four mechanical properties tests. A is the flexural property test sample with a length of 80±2 mm, a width of 15±0.5 mm, a thickness of 4±0.2 mm; B is the impact performance test sample, with a length of 80±2 mm, a width of 10±0.5 mm, a thickness of 4±0.2 mm; C is the toughness test sample, with a length of 60±2 mm, a width of 12±0.5 mm, a thickness of 6±0.2 mm; D is a DMA test spline with a length of 35±2 mm, a width of 8±0.2 mm and a thickness of 2±0.2 mm.

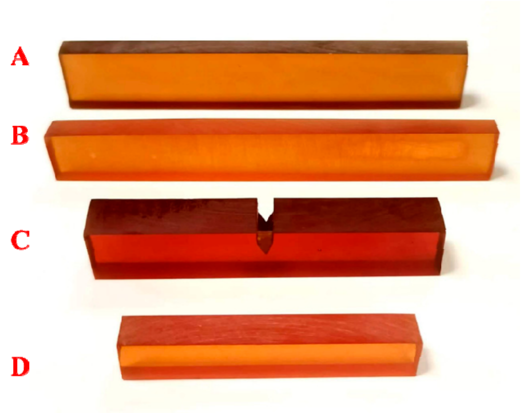

Figure S3. The sample diagram of four mechanical properties tests

A: flexural property test sample; B: impact performance test sample; C: toughness test sample; D: DMA test sample

Table S1. DSC analysis data of OMPPS-EP-i, OCOPS-EP-j, OGCPS-EP-k hybrid resins

Sample	T_i (°C)	T_p (°C)	T_f (°C)	ΔH (J/g)

EP	112.9	183.0	247.8	487.4
OMPPS-EP-0.3	110.9	181.8	247.3	541.4
OMPPS-EP-0.8	112.9	184.1	246.1	528.7
OMPPS-EP-1.4	111.5	184.7	246.4	538.7
OMPPS-EP-1.9	110.6	184.0	242.9	494.4
OMPPS-EP-2.8	107.8	183.1	245.2	529.6
OCOPS-EP-0.1	68.3	178.6	249.4	535.7
OCOPS-EP-0.3	67.6	171.9	248.4	582.9
OCOPS-EP-0.6	68.3	172.8	249.8	475.9
OCOPS-EP-0.8	67.4	175.3	247.2	523.3
OCOPS-EP-1.2	69.4	174.1	245.8	517.7
OGCPS-EP-0.1	117.2	190.4	248.3	518.2
OGCPS-EP-0.3	116.1	189.2	247.8	493.5
OGCPS-EP-0.6	116.1	190.0	247.5	498.5
OGCPS-EP-0.8	117.3	188.9	248.1	535.2
OGCPS-EP-1.2	113.2	188.8	248.1	506.3

[a] (a) the heating rate of the DSC analysis is 10 °C/min, (b) T_i is the initial peak curing temperature, T_p is the top curing peak temperature, T_f is the end curing peak temperature, and ΔH is the exothermic enthalpy of curing reaction.

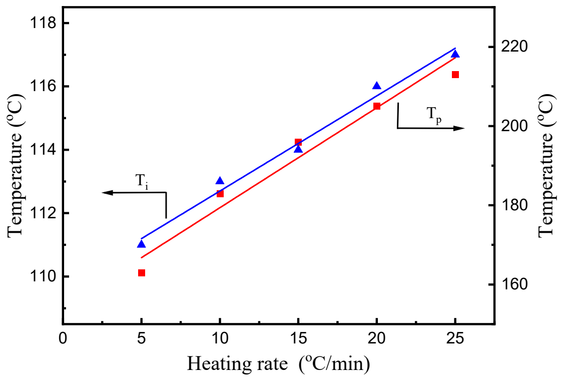

Figure S4. Linear fitting of T_i and T_p vs heating rate for the pristine resin

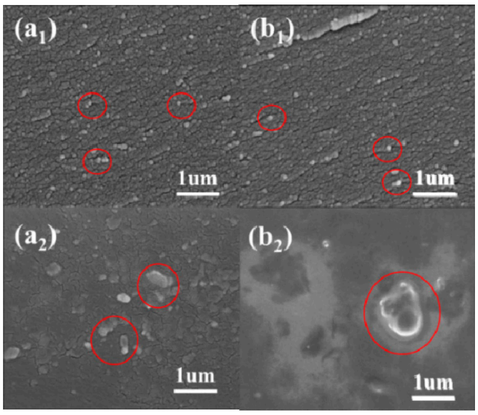

Figure S5. SEM images of fracture surface of OMPPS-EP-0.8-C (a_1), OMPPS-EP-1.9-C (a_2), OGCPS-EP-0.6-C (b_1), and OGCPS-EP-1.2 (b_2)

The crosslinking density of the polymer was calculated according to the formula S1[45].

$$d_c = \frac{E'}{3RT} \quad \text{S1}$$

Where d_c is the cross-linking density, unit mol/m^3; E' is the energy storage modulus of polymer at T_g+40°C, unit 0.1 Pa; R is the gas constant, 8.314 J/(mol·K); T is the absolute temperature, unit K. The calculated results are shown in Table S2. As shown in the table, the crosslinked density of the modified resins decreases with the addition amount of POSS modifier, which indicates the toughness of the modified resins rises.

Table S2. The cross-linking density of cured epoxy resins

Sample	d_c×10^4(mol/m^3)
EP	13.1
OMPPS-EP-0.3-C	9.8
OMPPS-EP-0.8-C	8.7
OMPPS-EP-1.4-C	9.3
OMPPS-EP-1.9-C	8.1
OMPPS-EP-2.8-C	10.2
OCOPS-EP-0.1-C	10.7
OCOPS-EP-0.3-C	8.7
OCOPS-EP-0.6-C	8.9
OCOPS-EP-0.8-C	8.8
OCOPS-EP-1.2-C	7.4
OGCPS-EP-0.1-C	10.8
OGCPS-EP-0.3-C	9.8
OGCPS-EP-0.6-C	9.7
OGCPS-EP-0.8-C	9.3
OGCPS-EP-1.2-C	10.4

45 Ohashi, S.; Kilbane, J.; Heyl, T.; Ishida, H. Synthesis and characterization of cyanate ester functional benzoxazine and its polymer. *Macromolecules*. **2015**, *48(23)*, 8412-8417.
